# Supplementary material for: S-nitrosylation of EZH2 alters PRC2 assembly, methyltransferase activity, and EZH2 stability to maintain endothelial homeostasis
Source: Nat Commun. 2025 Apr 27;16:3953. doi: 10.1038/s41467-025-59003-x (PMC12034783; doi:10.1038/s41467-025-59003-x)
Supplement: Supplementary file 1 — Supplementary Information [file 41467_2025_59003_MOESM1_ESM.pdf]

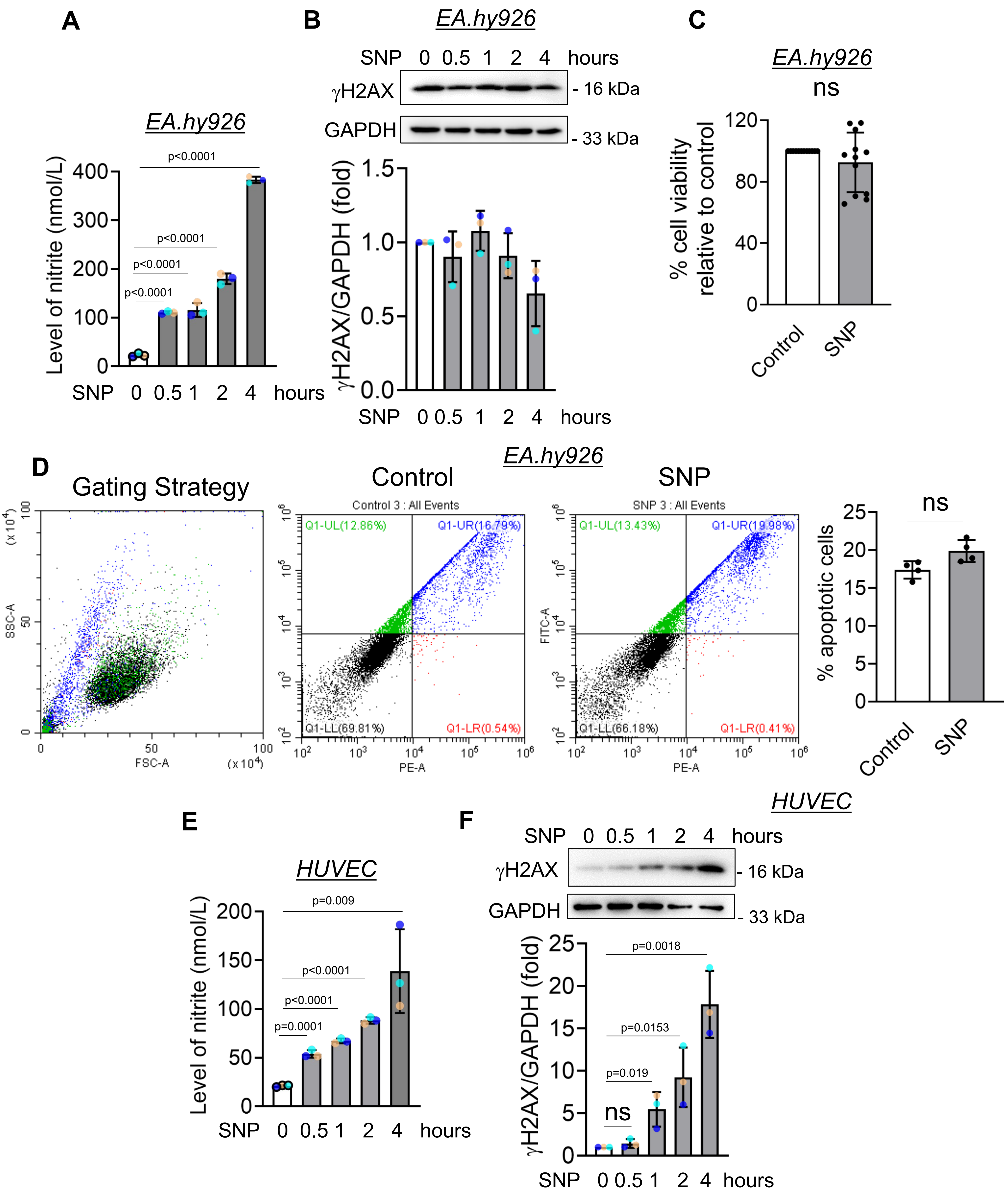
A
EA.hy926
Level of nitrite (nmol/L)
p<0.0001
p<0.0001
p<0.0001
p<0.0001
SNP 0 0.5 1 2 4 hours
B
EA.hy926
SNP 0 0.5 1 2 4 hours
γH2AX
- 16 kDa
GAPDH
- 33 kDa
γH2AX/GAPDH (fold)
SNP 0 0.5 1 2 4 hours
C
EA.hy926
ns
% cell viability relative to control
Control
SNP
D
Gating Strategy
EA.hy926
Control
SNP
Control 3 : All Events
SNP 3 : All Events
Q1-UL(12.86%)
Q1-UR(16.79%)
Q1-LL(69.81%)
Q1-LR(0.54%)
Q1-UL(13.43%)
Q1-UR(19.98%)
Q1-LL(66.18%)
Q1-LR(0.41%)
SSC-A
FSC-A
FITC-A
PE-A
ns
% apoptotic cells
Control
SNP
E
HUVEC
Level of nitrite (nmol/L)
p=0.009
p<0.0001
p<0.0001
p=0.0001
SNP 0 0.5 1 2 4 hours
F
HUVEC
SNP 0 0.5 1 2 4 hours
γH2AX
- 16 kDa
GAPDH
- 33 kDa
γH2AX/GAPDH (fold)
p=0.0018
p=0.0153
p=0.019
ns
SNP 0 0.5 1 2 4 hours

Supplementary Figure 1. Effect of SNP (500 μmol/L) on cellular nitrite level, DNA damage response gene, cell viability, and apoptosis in variety of endothelial cells. (A) The level of intracellular nitrite was measured using Griess Assay in EA.hy926 cells exposed to SNP (500 μM) for different tome points ranging from 0 to 4 hours. (n = 3, biological replicate) (B) Immunoblotting for γH2AX in EA.hy926 cells exposed to SNP (500 μM) for 0, 0.5, 1, 2, and 4 hours. (n = 3 , biological replicate) (C) Viability of EC treated with SNP (500 μM) for 24 hours was measured using MTT assay. (n = 12 , biological replicate) (D) First panel showed the gating strategy by defining the SSC and FSC of the cell population. Measuring the level of apoptosis in EA.hy926 cells exposed to SNP (500 μM) for 24 hours; distribution plots of early and late apoptotic cells and quantified data that include both early and late apoptotic cell population. (n = 4 , biological replicate). (E) The level of intracellular nitrite was measured using Griess Assay in HUVEC exposed to SNP (500 μM) for different tome points ranging from 0 to 4 hours. (n = 3 , biological replicate). (F) Immunoblotting for γH2AX in HUVEC cells exposed to SNP (500 μM) for 0, 0.5, 1, 2, and 4 hours. (n = 3 , biological replicate) All data are presented as mean values ± SD. All statistical analyses are either performed by One-way ANOVA with a post-hoc Tukey test for multiple groups or by two-tailed unpaired t-test for two groups.

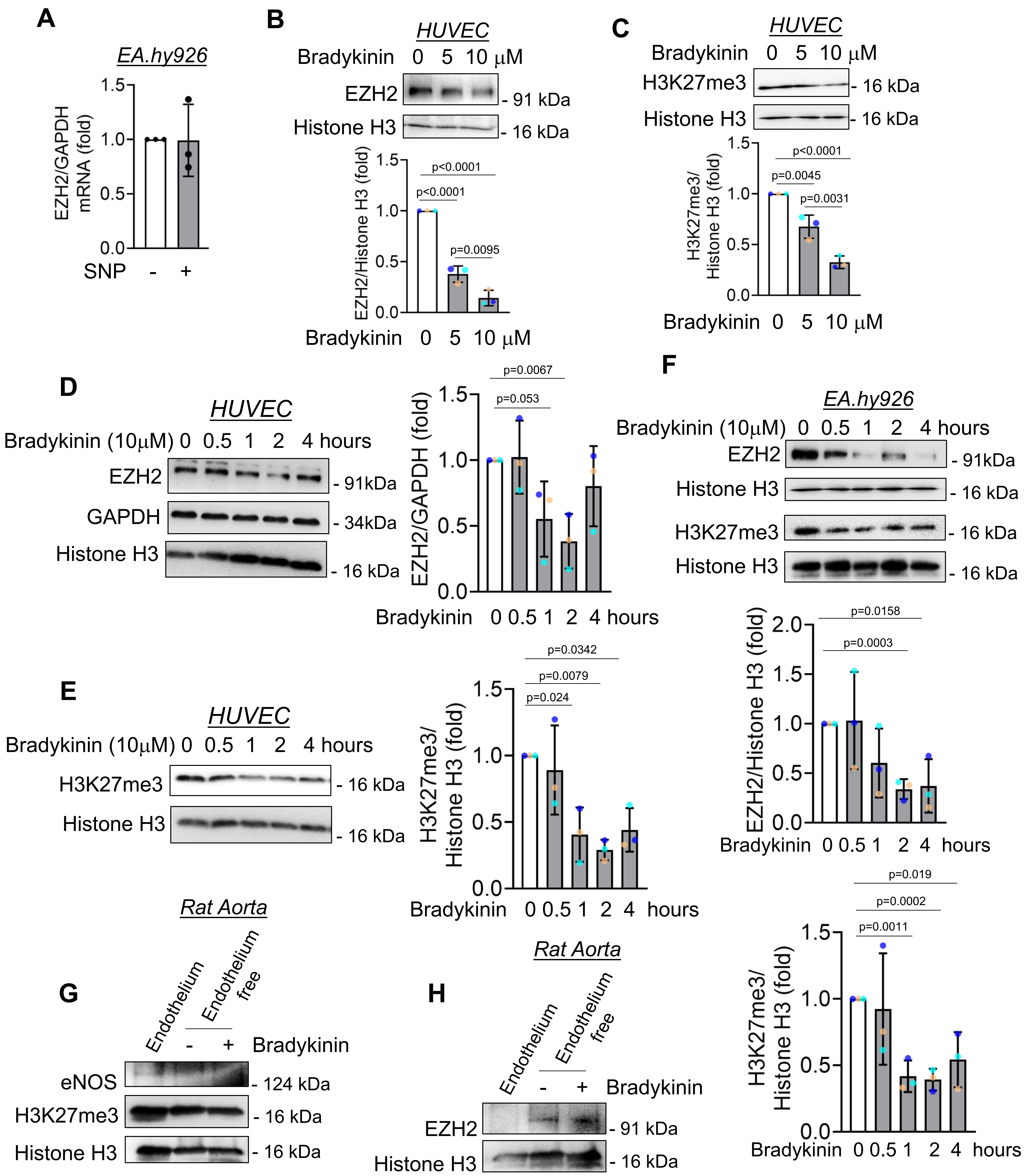

A
EA.hy926
EZH2/GAPDH mRNA (fold)
1.5
1.0
0.5
0.0
SNP - +
B
HUVEC
Bradykinin 0 5 10 μM
EZH2 - 91 kDa
Histone H3 - 16 kDa
EZH2/Histone H3 (fold)
p<0.0001
p<0.0001
p=0.0095
Bradykinin 0 5 10 μM
C
HUVEC
Bradykinin 0 5 10 μM
H3K27me3 - 16 kDa
Histone H3 - 16 kDa
H3K27me3/ Histone H3 (fold)
p<0.0001
p=0.0045
p=0.0031
Bradykinin 0 5 10 μM
D
HUVEC
Bradykinin (10μM) 0 0.5 1 2 4 hours
EZH2 - 91kDa
GAPDH - 34kDa
Histone H3 - 16 kDa
EZH2/GAPDH (fold)
p=0.0067
p=0.053
Bradykinin 0 0.5 1 2 4 hours
E
HUVEC
Bradykinin (10μM) 0 0.5 1 2 4 hours
H3K27me3 - 16 kDa
Histone H3 - 16 kDa
H3K27me3/ Histone H3 (fold)
p=0.0342
p=0.0079
p=0.024
Bradykinin 0 0.5 1 2 4 hours
F
EA.hy926
Bradykinin (10μM) 0 0.5 1 2 4 hours
EZH2 - 91kDa
Histone H3 - 16 kDa
H3K27me3 - 16 kDa
Histone H3 - 16 kDa
EZH2/Histone H3 (fold)
p=0.0158
p=0.0003
Bradykinin 0 0.5 1 2 4 hours
H3K27me3/ Histone H3 (fold)
p=0.019
p=0.0002
p=0.0011
Bradykinin 0 0.5 1 2 4 hours
G
Rat Aorta
Endothelium
Endothelium free
- + Bradykinin
eNOS - 124 kDa
H3K27me3 - 16 kDa
Histone H3 - 16 kDa
H
Rat Aorta
Endothelium
Endothelium free
- + Bradykinin
EZH2 - 91 kDa
Histone H3 - 16 kDa

Supplementary Figure 2. Induction of endogenous nitric oxide or nitric oxide supplementation altered EZH2 and H3K27me3 levels independent of cell types and without altering EZH2 transcript level. (A) qPCR analysis to measure the transcript level of EZH2 in cultured EA.hy926 cells exposed to SNP (500 μM) for 2 hours. (n=3 , biological replicate) (B-C) Immunoblotting for EZH2 (B) and H3K27me3 (C) in HUVEC exposed to different concentrations of bradykinin (0, 5, and 10 μM) for 2 hours. (n = 3 , biological replicate) (D-F) Immunoblotting for EZH2 (D,F) and H3K27me3 (E,F) in HUVEC (D, E, n = 3 , biological replicate) or EA.hy926 cells (F, n = 3 , biological replicate) exposed to bradykinin (10 μM) for different time points (0, 0.5, 1, 2, and 4 hours). (G-H) Immunoblot analysis using lysate of rat aortic explants (with or without endothelial layer) exposed to bradykinin (10 μM) for 2 hours. (n = 3 , biological replicate) All data are presented as mean values ± SD. All statistical analyses are either performed by One-way ANOVA with a post-hoc Tukey test for multiple groups or by two-tailed unpaired t-test for two groups.

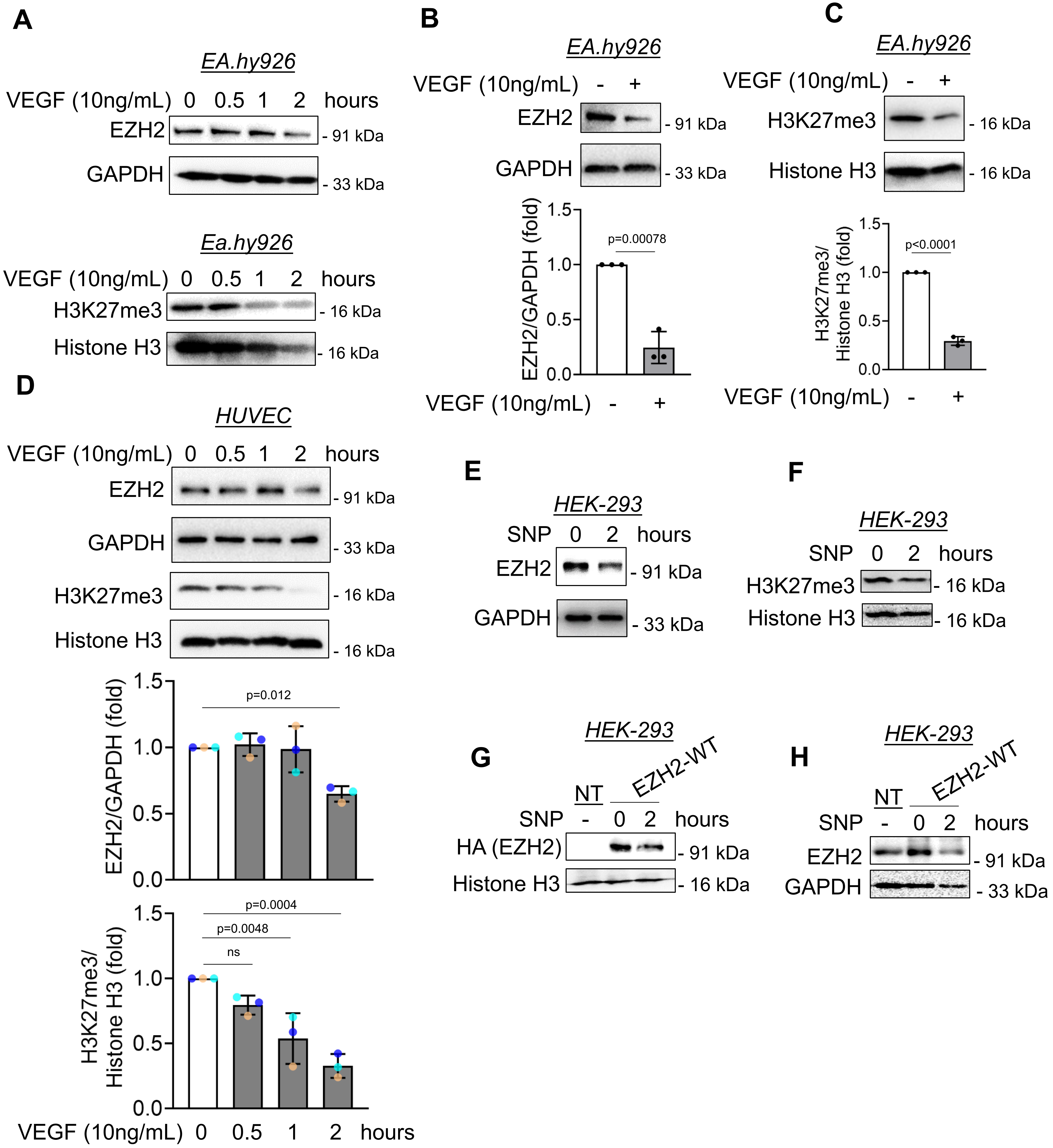

A
EA.hy926
VEGF (10ng/mL) 0 0.5 1 2 hours
EZH2
- 91 kDa
GAPDH
- 33 kDa
Ea.hy926
VEGF (10ng/mL) 0 0.5 1 2 hours
H3K27me3
- 16 kDa
Histone H3
- 16 kDa
B
EA.hy926
VEGF (10ng/mL) - +
EZH2
- 91 kDa
GAPDH
- 33 kDa
EZH2/GAPDH (fold)
p=0.00078
VEGF (10ng/mL) - +
C
EA.hy926
VEGF (10ng/mL) - +
H3K27me3
- 16 kDa
Histone H3
- 16 kDa
H3K27me3/ Histone H3 (fold)
p<0.0001
VEGF (10ng/mL) - +
D
HUVEC
VEGF (10ng/mL) 0 0.5 1 2 hours
EZH2
- 91 kDa
GAPDH
- 33 kDa
H3K27me3
- 16 kDa
Histone H3
- 16 kDa
EZH2/GAPDH (fold)
p=0.012
H3K27me3/ Histone H3 (fold)
p=0.0004
p=0.0048
ns
VEGF (10ng/mL) 0 0.5 1 2 hours
E
HEK-293
SNP 0 2 hours
EZH2
- 91 kDa
GAPDH
- 33 kDa
F
HEK-293
SNP 0 2 hours
H3K27me3
- 16 kDa
Histone H3
- 16 kDa
G
HEK-293
NT EZH2-WT
SNP - 0 2 hours
HA (EZH2)
- 91 kDa
Histone H3
- 16 kDa
H
HEK-293
NT EZH2-WT
SNP - 0 2 hours
EZH2
- 91 kDa
GAPDH
- 33 kDa

Supplementary Figure 3. Induction with VEGF or nitric oxide supplementation altered EZH2 and H3K27me3 levels in endothelial cells or HEK-293 respectively. (A) Immunoblotting for EZH2 and H3K27me3 in EA.hy926 cells exposed to vascular endothelial growth factor (VEGF) (10 ng/mL) for different time points (0, 0.5, 1, and 2 hours). (n = 3 , biological replicate) (B-C) EZH2 (B) and H3K27me3 (C) were measured using immunoblot assay with EC induced with VEGF (10 ng/mL) for 2 hours. (n = 3 , biological replicate) (D) Immunoblotting for EZH2 and H3K27me3 in HUVEC exposed to vascular endothelial growth factor (VEGF) (10 ng/mL) for different time points (0, 0.5, 1, and 2 hours). (n = 3 , biological replicate) (E-F) Immunoblotting for EZH2 (E) and H3K27me3 (F) in HEK-293 cells on exposure to SNP (500 μM) for 2 hours. (n = 3 , biological replicate) (G-H) Immunoblotting experiment to show the HA-EZH2 (G) and EZH2 (H) levels in the lysates collected from HEK-293 cells transfected with plasmid containing HA tagged EZH2 followed by treatment with SNP (500 μM) for 2 hours. (n = 3 , biological replicate) All data are presented as mean values ± SD. All statistical analyses are either performed by One-way ANOVA with a post-hoc Tukey test for multiple groups or by two-tailed unpaired t-test for two groups.

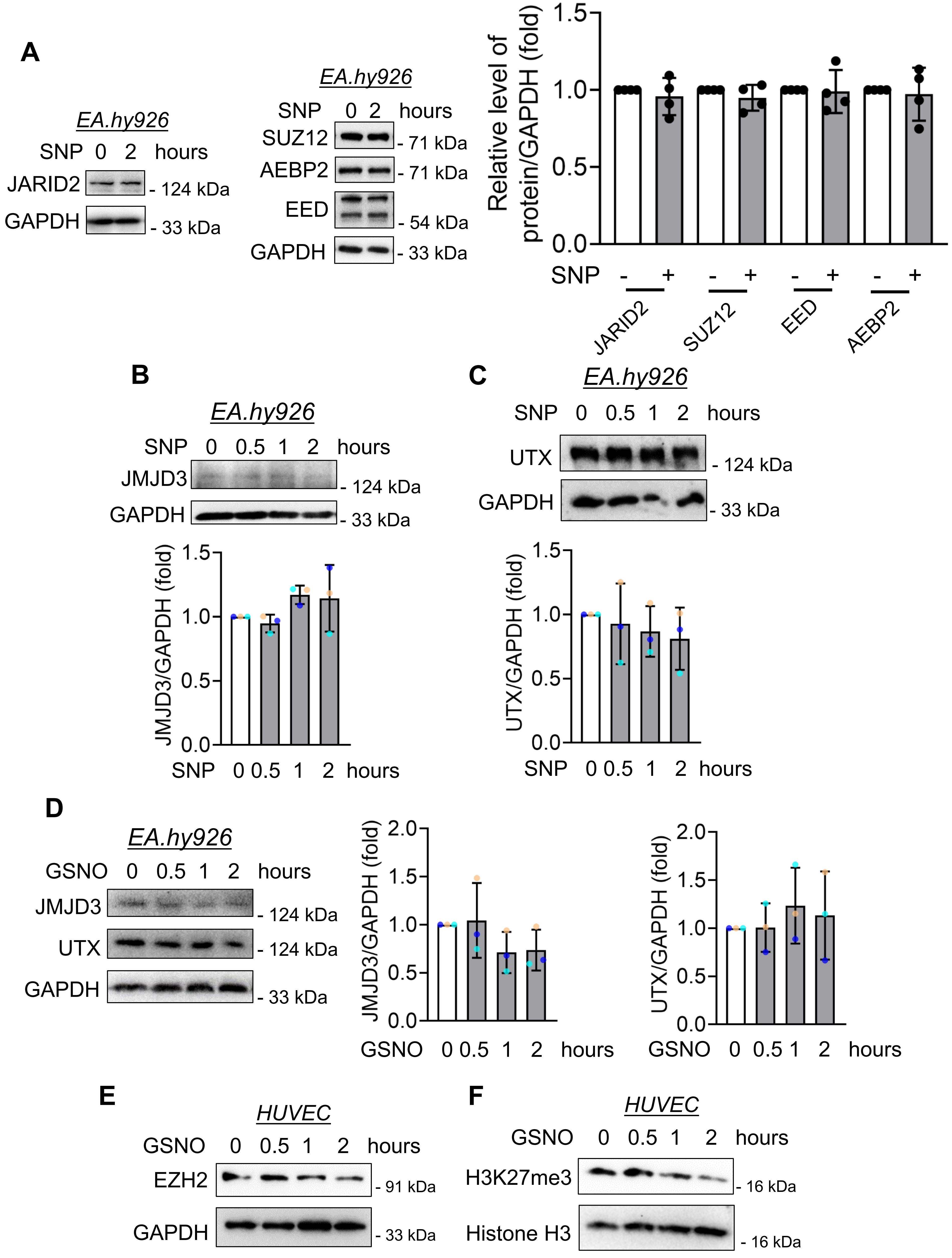

A
EA.hy926
SNP 0 2 hours
JARID2 - 124 kDa
GAPDH - 33 kDa
EA.hy926
SNP 0 2 hours
SUZ12 - 71 kDa
AEBP2 - 71 kDa
EED - 54 kDa
GAPDH - 33 kDa
Relative level of protein/GAPDH (fold)
1.5
1.0
0.5
0.0
SNP - + - + - + - +
JARID2
SUZ12
EED
AEBP2
B
EA.hy926
SNP 0 0.5 1 2 hours
JMJD3 - 124 kDa
GAPDH - 33 kDa
JMJD3/GAPDH (fold)
1.5
1.0
0.5
0.0
SNP 0 0.5 1 2 hours
C
EA.hy926
SNP 0 0.5 1 2 hours
UTX - 124 kDa
GAPDH - 33 kDa
UTX/GAPDH (fold)
1.5
1.0
0.5
0.0
SNP 0 0.5 1 2 hours
D
EA.hy926
GSNO 0 0.5 1 2 hours
JMJD3 - 124 kDa
UTX - 124 kDa
GAPDH - 33 kDa
JMJD3/GAPDH (fold)
2.0
1.5
1.0
0.5
0.0
GSNO 0 0.5 1 2 hours
UTX/GAPDH (fold)
2.0
1.5
1.0
0.5
0.0
GSNO 0 0.5 1 2 hours
E
HUVEC
GSNO 0 0.5 1 2 hours
EZH2 - 91 kDa
GAPDH - 33 kDa
F
HUVEC
GSNO 0 0.5 1 2 hours
H3K27me3 - 16 kDa
Histone H3 - 16 kDa

Supplementary Figure 4. SNP did not alter the level of other PRC2 complex proteins and UTX and JMJD3, H3K27me3 specific demethylases remained unchanged in EA.hy926 cells exposed to SNP or GSNO while GSNO temporally reduced the level of EZH2 and H3K27me3 in HUVEC. (A) Immunoblotting for SUZ12, AEBP2, JARID2, and EED after exposure of EA.hy926 cells to NO donor SNP (500 μM) for 2 hours. (n = 4 , biological replicate) (B-D) Immunoblotting for JMJD3 (B,D) and UTX (C,D) after exposing EA.hy926 cells to SNP (B,C) (500 μM) or GSNO (D) (100 μM) for different time points (0, 0.5, 1, and 2 hours). (n = 3 , biological replicate) (E-F) Immunoblotting for EZH2 and H3K27me3 in cultured HUVEC exposed to GSNO (100 μM) for different time points (0, 0.5, 1, and 2 hours). (n = 3 , biological replicate) All data are presented as mean values ± SD. All statistical analyses are either performed by One-way ANOVA with a post-hoc Tukey test for multiple groups or by two-tailed unpaired t-test for two groups.

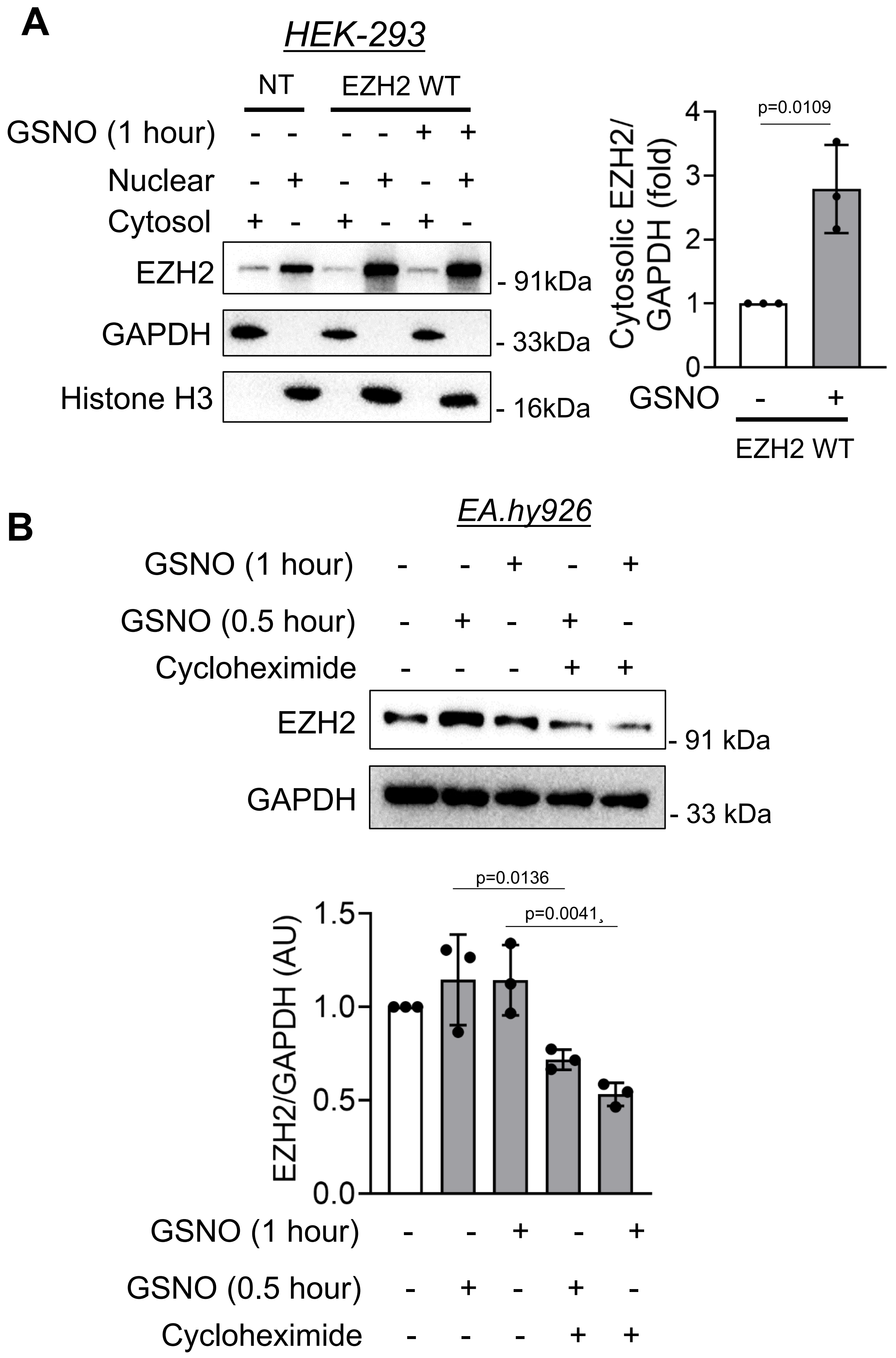
A
HEK-293
NT
EZH2 WT
GSNO (1 hour)
Nuclear
Cytosol
EZH2
GAPDH
Histone H3
- 91kDa
- 33kDa
- 16kDa
Cytosolic EZH2/ GAPDH (fold)
p=0.0109
GSNO
EZH2 WT
B
EA.hy926
GSNO (1 hour)
GSNO (0.5 hour)
Cycloheximide
EZH2
GAPDH
- 91 kDa
- 33 kDa
EZH2/GAPDH (AU)
p=0.0136
p=0.0041

Supplementary Figure 5. GSNO caused cytosolic localization of overexpressed EZH2 WT in HEK-293 cells while the cycloheximide chase experiment revealed early degradation of EZH2 when EZH2 protein turnover was blocked by inhibiting translation. (A) Sub-cellular fractionation followed by immunoblotting of cell lysates collected from HEK-293 cells transfected with EZH2 WT containing plasmid and challenged with GSNO (100 μM) for 1 hour. EZH2 was detected in the given immunoblot along with detecting the presence of GAPDH and histone H3 only in cytosolic and nuclear fractions respectively to indicate the purity of the cellular fractions. (n = 3 , biological replicate) (B) EZH2 was detected in EA.hy926 cells pretreated with cycloheximide (10 μg/mL) for 4 hours followed by exposing to GSNO (100 μM) for different time points (0, 0.5, and 1 hour). (n = 3 , biological replicate) All data are presented as mean values ± SD. All statistical analyses are either performed by One-way ANOVA with a post-hoc Tukey test for multiple groups or by two-tailed unpaired t-test for two groups.

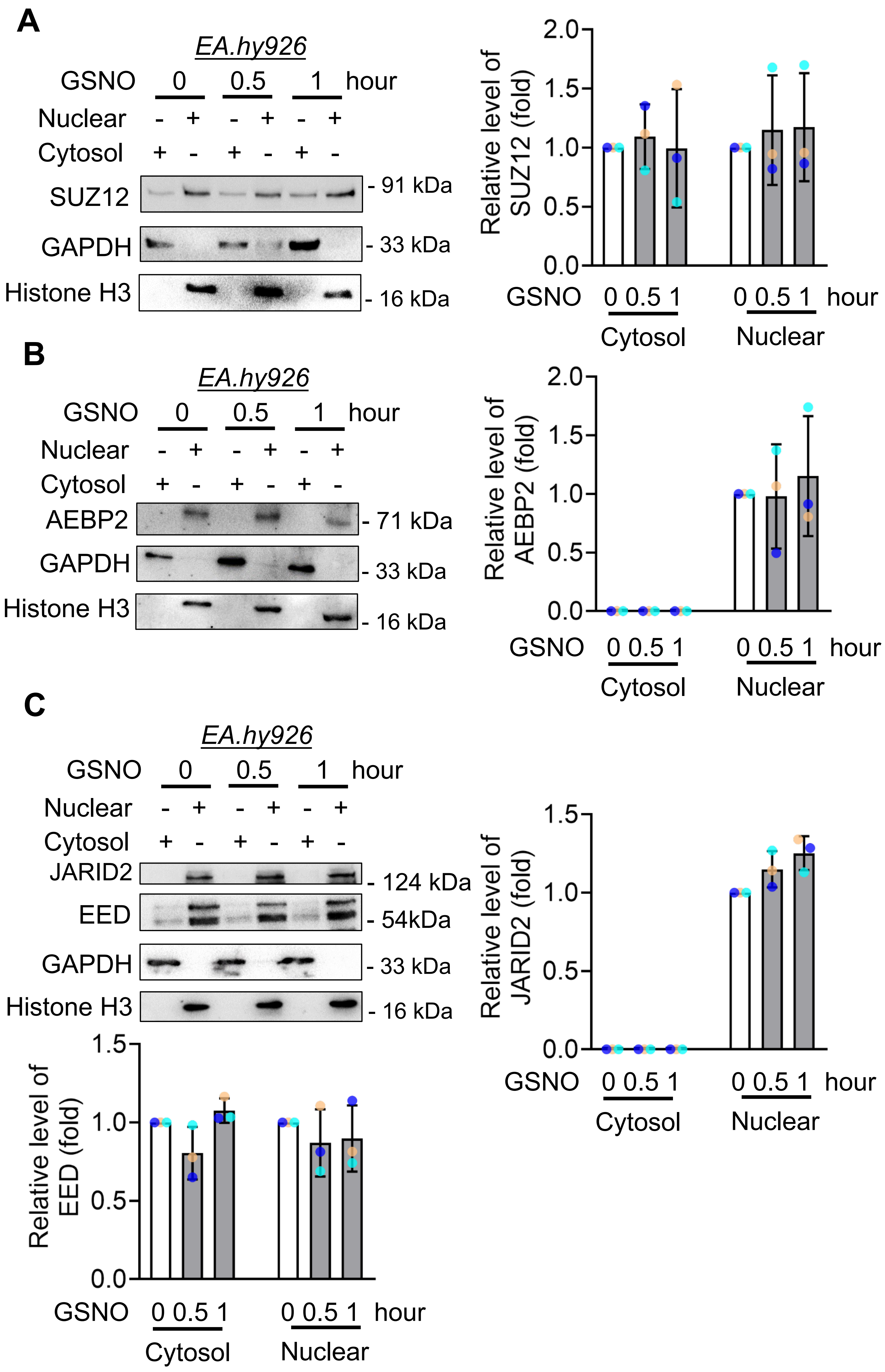
A
EA.hy926
GSNO 0 0.5 1 hour
Nuclear - + - + - +
Cytosol + - + - + -
SUZ12
GAPDH
Histone H3
- 91 kDa
- 33 kDa
- 16 kDa
Relative level of SUZ12 (fold)
2.0
1.5
1.0
0.5
0.0
GSNO 0 0.5 1 0 0.5 1 hour
Cytosol
Nuclear
B
EA.hy926
GSNO 0 0.5 1 hour
Nuclear - + - + - +
Cytosol + - + - + -
AEBP2
GAPDH
Histone H3
- 71 kDa
- 33 kDa
- 16 kDa
Relative level of AEBP2 (fold)
2.0
1.5
1.0
0.5
0.0
GSNO 0 0.5 1 0 0.5 1 hour
Cytosol
Nuclear
C
EA.hy926
GSNO 0 0.5 1 hour
Nuclear - + - + - +
Cytosol + - + - + -
JARID2
EED
GAPDH
Histone H3
- 124 kDa
- 54kDa
- 33 kDa
- 16 kDa
Relative level of JARID2 (fold)
1.5
1.0
0.5
0.0
GSNO 0 0.5 1 0 0.5 1 hour
Cytosol
Nuclear
Relative level of EED (fold)
1.5
1.0
0.5
0.0
GSNO 0 0.5 1 0 0.5 1 hour
Cytosol
Nuclear

Supplementary Figure 6. GSNO challenge did not alter the intracellular localization of other PRC2 complex proteins. (A-C) Sub-cellular fractionation of cell lysates collected from EA.hy926 cells exposed to GSNO (100 μM) for 0.5 and 1 hour followed by immunoblotting experiment for SUZ12 (A), AEBP2 (B), JARID2 (C), and EED (C). GAPDH and histone H3 were used to confirm the cytosolic and nuclear fractions respectively. (n = 3 , biological replicate) All data are presented as mean values ± SD. All statistical analyses are either performed by One-way ANOVA with a post-hoc Tukey test for multiple groups or by two-tailed unpaired t-test for two groups.

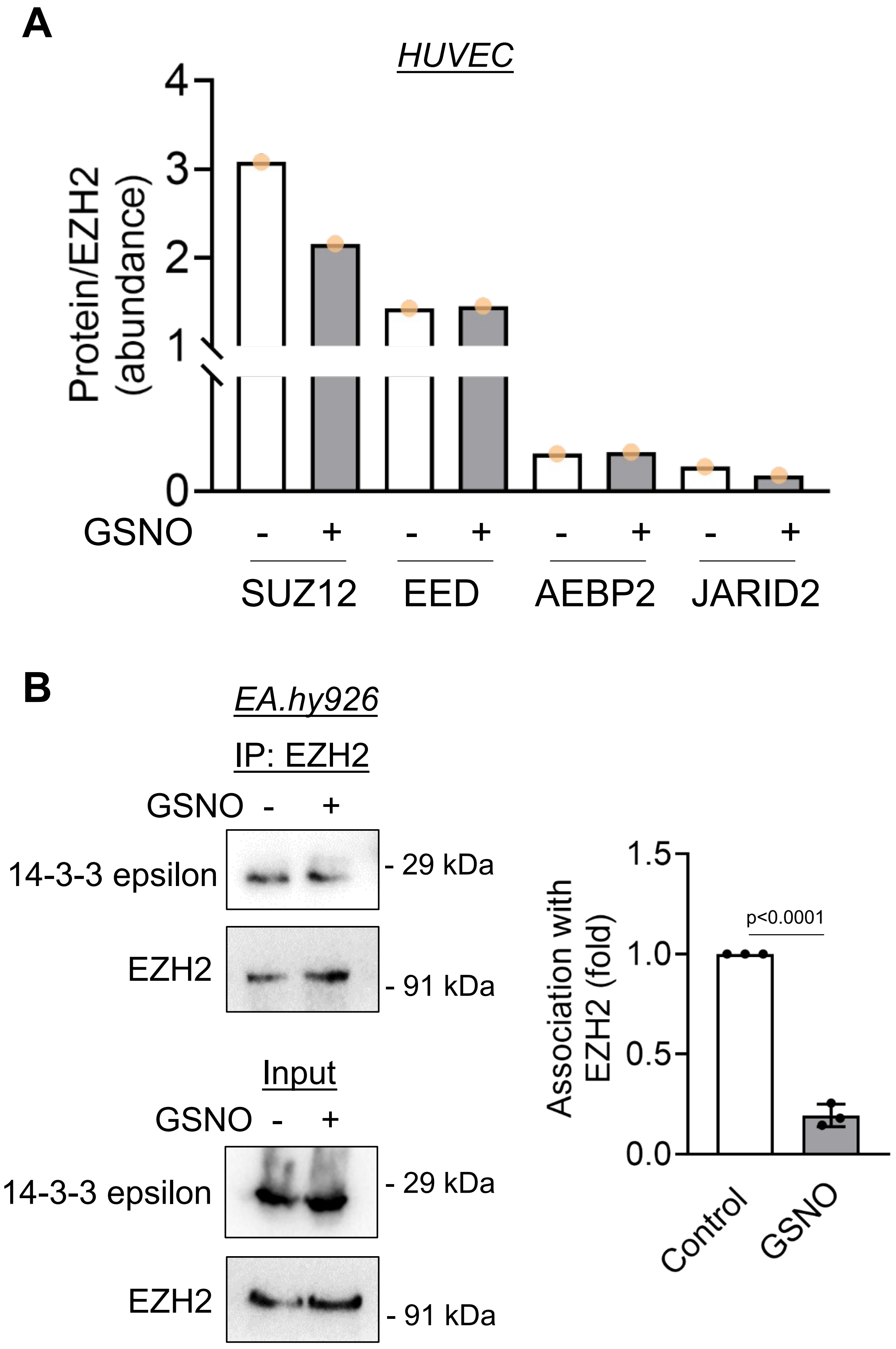

Supplementary Figure 7. GSNO caused dissociation of certain PRC2 proteins and 14-3-3ε from EZH2. (A) Mass spectroscopy based abundance analysis of EZH2 interacting partners of PRC2 complex including SUZ12, EED, AEBP2, and JARID2. Data was normalized to the relative abundance of EZH2. (n = 2, biological replicate) (B) EA.hy926 cells treated with GSNO (100 μM) for 0.5 hour were subjected to co-immunoprecipitation using EZH2 antibody followed by immunoblotting to show the association of EZH2 with 14-3-3ε. (n = 3, biological replicate) All data are presented as mean values ± SD. All statistical analyses are either performed by two-tailed unpaired t-test for two groups.

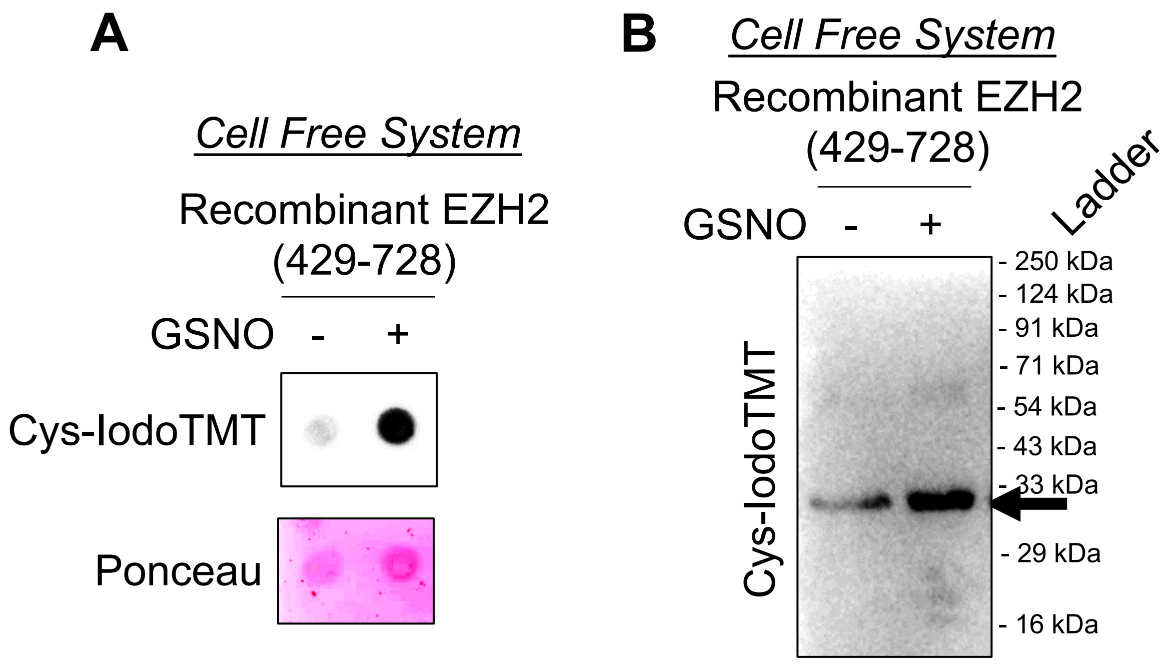

Supplementary Figure 8. Confirming S-nitrosylation of recombinant human EZH2 (containing aa 429-728) using IdodTMT assay in a cell free system. (A-B) In a cell free system, a total of 1 µg recombinant human EZH2 (containing aa 429-728) protein was incubated with GSNO (100 µM) for 30 minutes followed by processing through iodoTMT protocol. Dot blots were carried out and incubated with anti-IodoTMT antibody. Blots were developed with chemiluminescence substrate for visualization (A). Samples were run through SDS-PAGE followed by transferring to nitrocellulose membrane and were incubated with anti-IodoTMT antibody. Blots were developed with chemiluminescence substrate for visualization (B). (n=3, biological replicate)

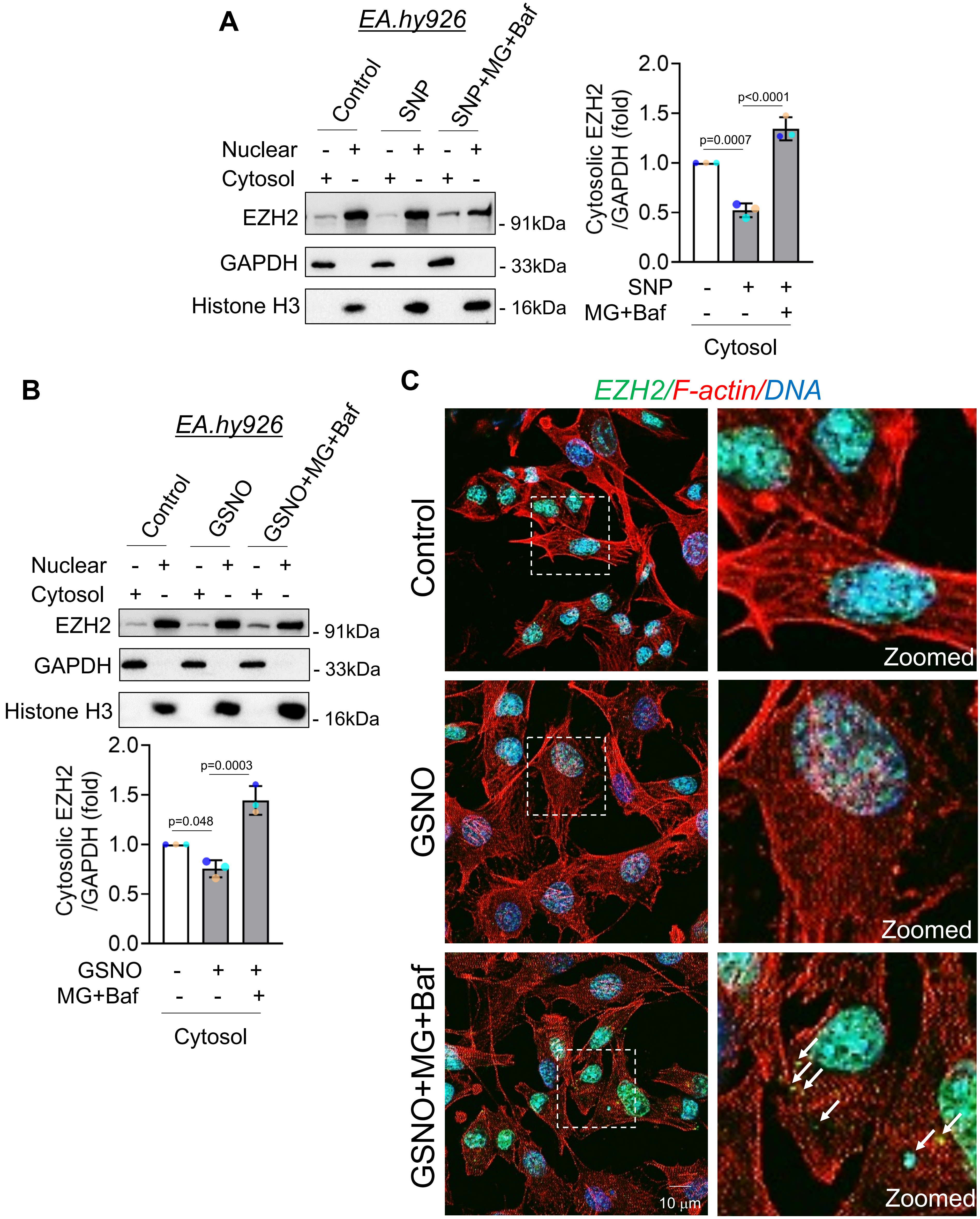
A
EA.hy926
Control
SNP
SNP+MG+Baf
Nuclear - + - + - +
Cytosol + - + - + -
EZH2
- 91kDa
GAPDH
- 33kDa
Histone H3
- 16kDa
Cytosolic EZH2 /GAPDH (fold)
2.0
1.5
1.0
0.5
0.0
p=0.0007
p<0.0001
SNP - + +
MG+Baf - - +
Cytosol
B
EA.hy926
Control
GSNO
GSNO+MG+Baf
Nuclear - + - + - +
Cytosol + - + - + -
EZH2
- 91kDa
GAPDH
- 33kDa
Histone H3
- 16kDa
Cytosolic EZH2 /GAPDH (fold)
2.0
1.5
1.0
0.5
0.0
p=0.048
p=0.0003
GSNO - + +
MG+Baf - - +
Cytosol
C
EZH2/F-actin/DNA
Control
GSNO
GSNO+MG+Baf
Zoomed
Zoomed
Zoomed
10 µm

Supplementary Figure 9. Blocking the degradation pathways caused cytosolic accumulation of EZH2 in EC exposed to SNP or GSNO. (A-B) Immunoblot analysis of sub-cellular fractionation sample of cell lysates collected from HEK-293 cells pretreated with both MG-132 (MG) (1 μM) and bafilomycinA1 (Baf) (100 nM) for 2 hours followed by exposing to SNP (A, 500 μM) or GSNO (B, 100 μM) for additional 2 hours. EZH2 was detected in these immunoblots to analyze its relative abundance in cytosolic and nuclear fractions. GAPDH and histone H3 were used to confirm the cytosolic and nuclear fractions respectively. (n = 3 , biological replicate) (C) Immunofluorescence followed by confocal imaging of EA.hy926 cells pretreated with both MG-132 (MG) (1 μM) and bafilomycinA1 (Baf) (100 nM) for 2 hours followed by exposing to GSNO (100 μM) for additional 2 hours to show the cytosolic translocation of EZH2 (green). F-actin (red) is stained with phalloidin-Alexa Fluor 555. DAPI staining is shown in blue. (Scale bar: 10 μm). (n = 3 , images are the representative of three biological replicate). All data are presented as mean values ± SD. All statistical analyses are either performed by One-way ANOVA with a post-hoc Tukey test for multiple groups or by two-tailed unpaired t-test for two groups.

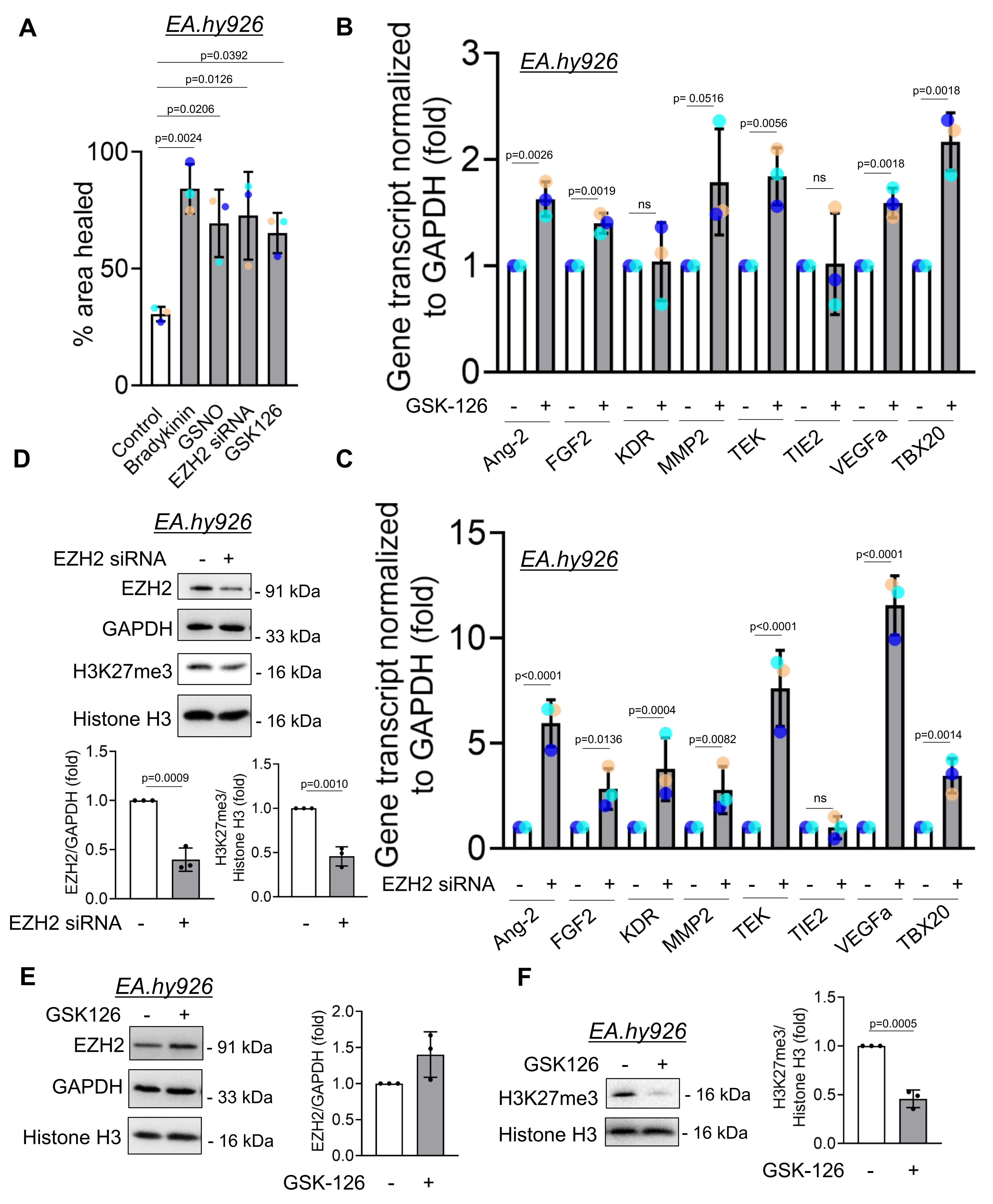
A
EA.hy926
p=0.0392
p=0.0126
p=0.0206
p=0.0024
% area healed
100
50
0
Control
Bradykinin
GSNO
EZH2 siRNA
GSK126
B
EA.hy926
Gene transcript normalized to GAPDH (fold)
3
2
1
0
p=0.0026
p=0.0019
ns
p= 0.0516
p=0.0056
ns
p=0.0018
p=0.0018
GSK-126
- + - + - + - + - + - + - + - +
Ang-2
FGF2
KDR
MMP2
TEK
TIE2
VEGFa
TBX20
C
EA.hy926
Gene transcript normalized to GAPDH (fold)
15
10
5
0
p<0.0001
p=0.0136
p=0.0004
p=0.0082
p<0.0001
ns
p<0.0001
p=0.0014
EZH2 siRNA
- + - + - + - + - + - + - + - +
Ang-2
FGF2
KDR
MMP2
TEK
TIE2
VEGFa
TBX20
D
EA.hy926
EZH2 siRNA - +
EZH2 - 91 kDa
GAPDH - 33 kDa
H3K27me3 - 16 kDa
Histone H3 - 16 kDa
EZH2/GAPDH (fold)
1.5
1.0
0.5
0.0
p=0.0009
H3K27me3/ Histone H3 (fold)
p=0.0010
EZH2 siRNA - + - +
E
EA.hy926
GSK126 - +
EZH2 - 91 kDa
GAPDH - 33 kDa
Histone H3 - 16 kDa
EZH2/GAPDH (fold)
2.0
1.5
1.0
0.5
0.0
GSK-126 - +
F
EA.hy926
GSK126 - +
H3K27me3 - 16 kDa
Histone H3 - 16 kDa
H3K27me3/ Histone H3 (fold)
1.5
1.0
0.5
0.0
p=0.0005
GSK-126 - +

Supplementary Figure 10. Interplaying with the EZH2-H3K27me3 axis by S-nitrosylation inducing agents or by pharmacological inhibitor or by knockdown technique resulted in comparable changes in endothelial migration and gene expression. (A) Scratch wound healing assay to show the healing rate of EA.hy926 cells exposed to bradykinin (10 µM) or GSNO (100 µM) or EZH2 siRNA (40 nM, transfected using lipofectamine 2000 reagent) or GSK126 (10 µM). Healing rate was followed until 24 hours after treatment. Images were acquired using bright field microscope adapted with a camera for phase contrast imaging. Healing rate was analyzed by calculating the area of the wound at 0 and 24 hours using Image J software. (n = 3 , biological replicate) (B-C) RT-qPCR analysis to measure the transcript level expression of *VEGFa*, *TBX20*, *MMP2*, *FGF2*, *KDR*, *TIE2*, *TEK*, and *Angiopoietin-2* in EA.hy926 cells treated with GSK126 (B, 10 µM) for 24 hours or transfected with EZH2 siRNA (C, 40 nM) and incubated for 24 hours. (n = 3 , biological replicate) (D-F) Immunoblotting for EZH2 (D,E) and H3K27me3 (D,F) in EA.hy926 cells transfected with EZH2 siRNA (D, 40 nM) or exposed to GSK126 (E-F, 10 µM) for 24 hours or and incubated for 24 hours. (n = 3 , biological replicate) All data are presented as mean values ± SD. All statistical analyses are either performed by One-way ANOVA with a post-hoc Tukey test for multiple groups or by two-tailed unpaired t-test for two groups.

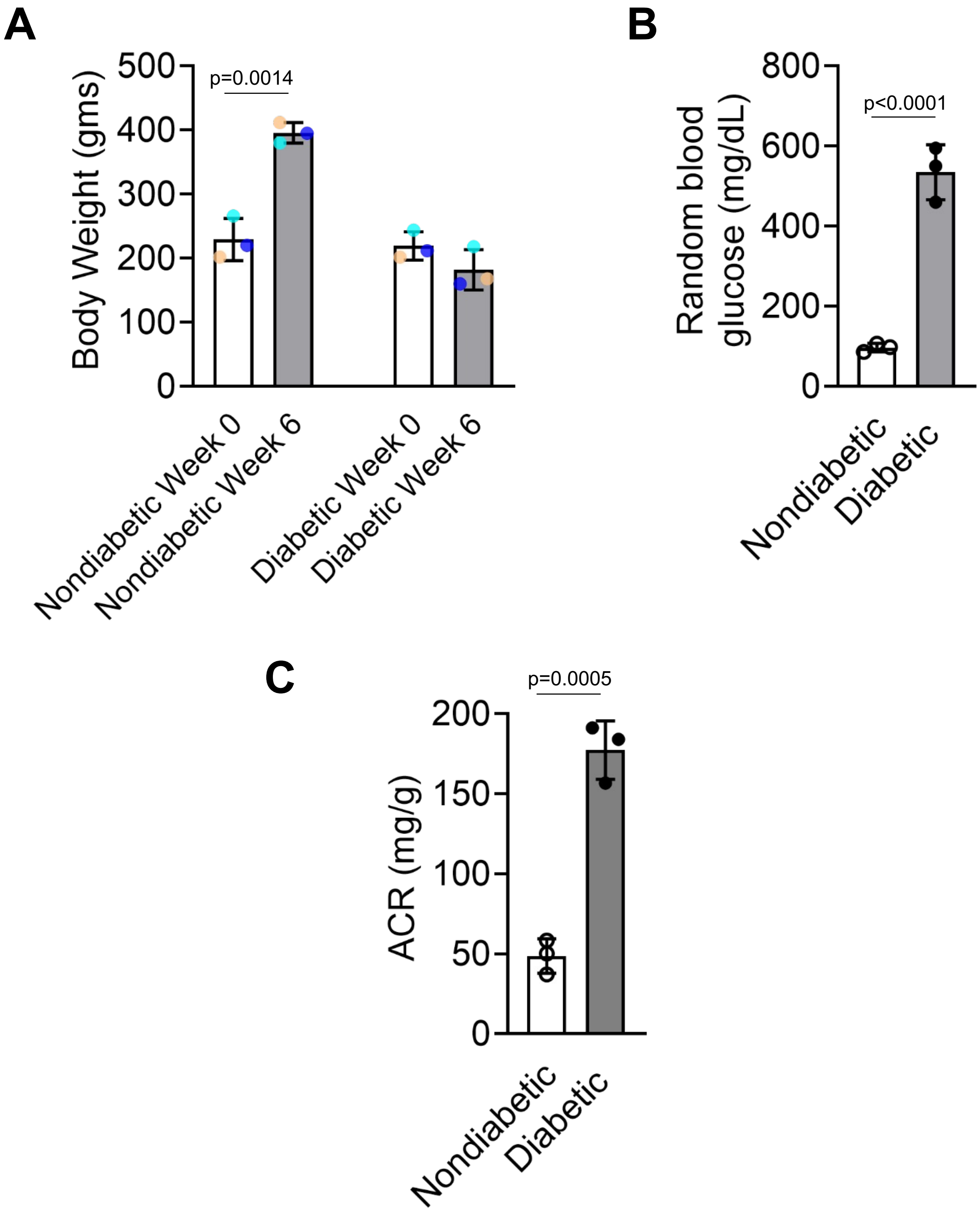

Supplemental Figure 11. Different parameters of rat undergone STZ treatment to develop diabetic kidney disease. (A) Body weight of the rats at Week 0 prior to STZ and 6 weeks post STZ treatment. (B) Random blood glucose level of nondiabetic and diabetic rats 6 weeks post STZ treatment. (C) Albumin-to-creatinine ratio to confirm diabetic kidney disease. (n = 3, biological replicate) All data are presented as mean values ± SD. All statistical analyses are either performed by One-way ANOVA with a post-hoc Tukey test for multiple groups or by two-tailed unpaired t-test for two groups.

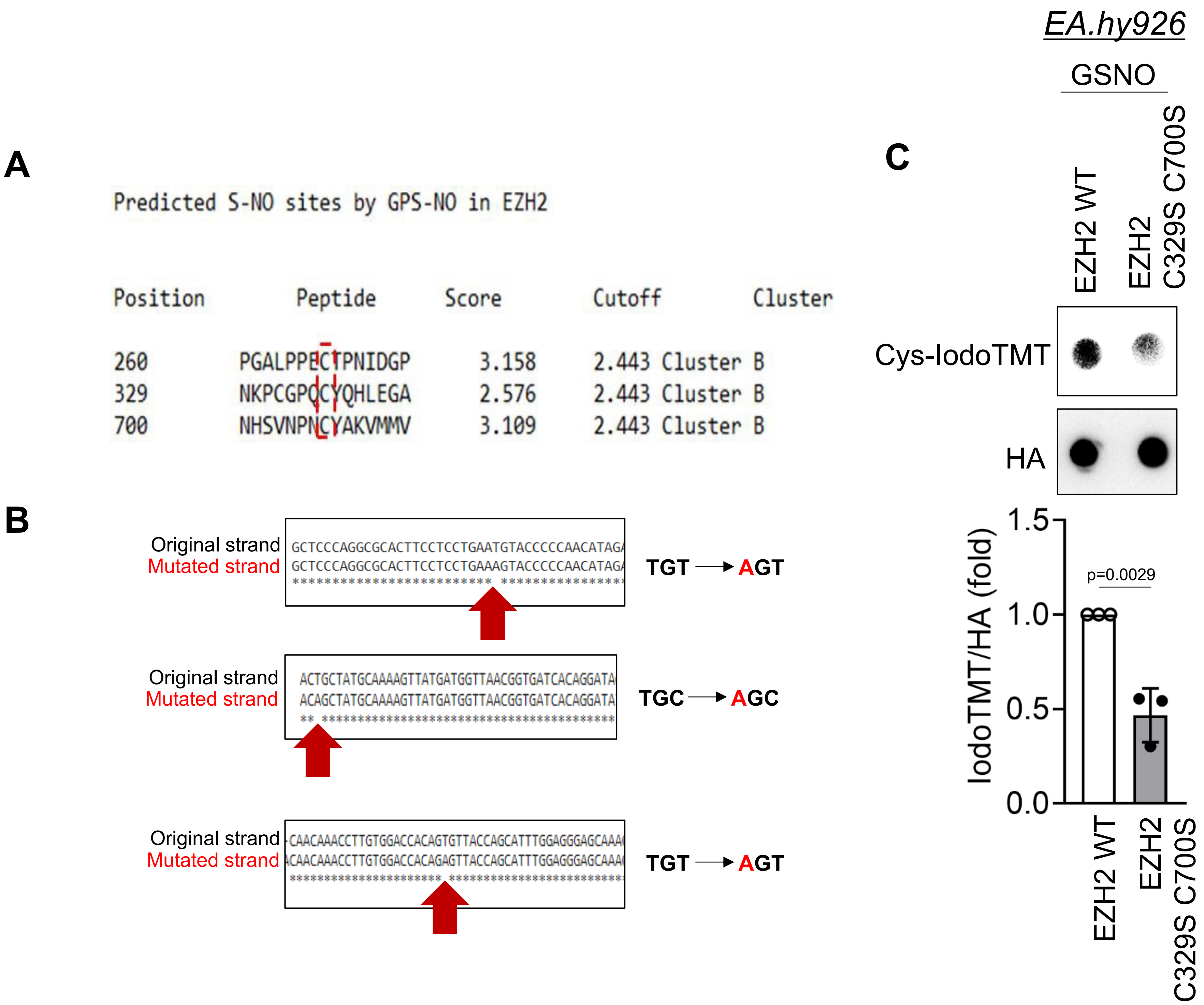

Supplementary Figure 12. *In silico* S-nitrosylation prediction analysis and generating point mutants for identified residues using site directed mutagenesis kit. (A) Using a pre-validated software, we performed an *in silico* analysis to predict possible cysteine residues that could be S-nitrosylated and identified three cysteine residues at position 260, 329 and 700 (out of 34 total cysteine present in EZH2) that were predicted to be S-nitrosylated. (B) Confirming the insertion of point mutations at these three predicted cysteine sites leading to their conversion to serine using Sanger sequencing technique. (C) Dot blot of protein lysates collected from EA.hy926 cells exposed to GSNO (100 μM) for 0.5 hour followed by immunoprecipitation with HA antibody and processing through iodoTMT protocol. Blots were incubated with anti-IodoTMT antibody. Blots were developed with chemiluminescence substrate for visualization. (n = 3 , biological replicate) All data are presented as mean values ± SD. All statistical analyses are either performed by One-way ANOVA with a post-hoc Tukey test for multiple groups or by two-tailed unpaired t-test for two groups.

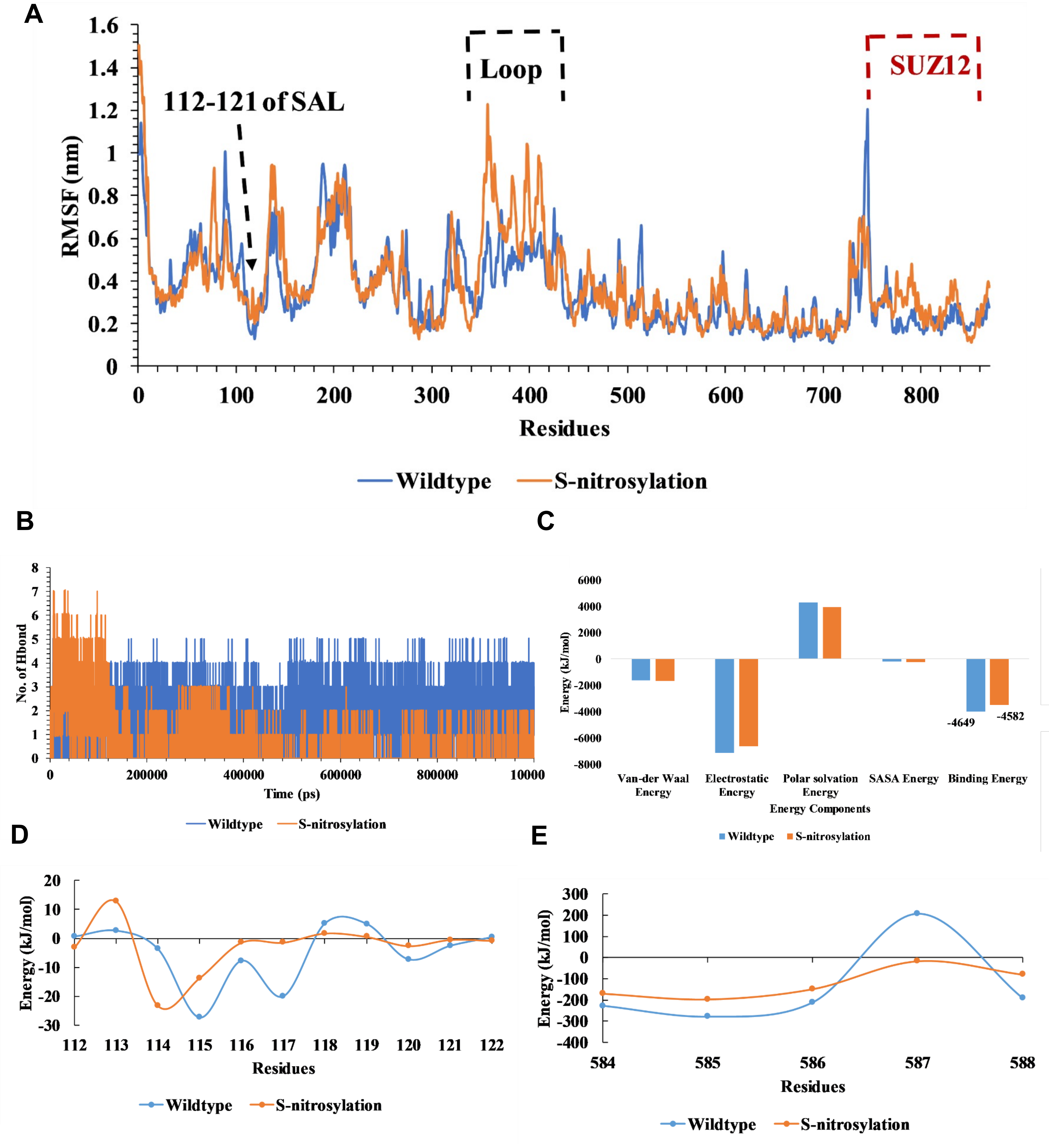
A
1.6
1.4
1.2
1
0.8
0.6
0.4
0.2
0
RMSF (nm)
112-121 of SAL
Loop
SUZ12
0
100
200
300
400
500
600
700
800
Residues
Wildtype
S-nitrosylation
B
8
7
6
5
4
3
2
1
0
No. of Hbond
0
200000
400000
600000
800000
10000
Time (ps)
Wildtype
S-nitrosylation
C
6000
4000
2000
0
-2000
-4000
-6000
-8000
Energy (kJ/mol)
-4649
-4582
Van-der Waal Energy
Electrostatic Energy
Polar solvation Energy
SASA Energy
Binding Energy
Energy Components
Wildtype
S-nitrosylation
D
20
10
0
-10
-20
-30
Energy (kJ/mol)
112
113
114
115
116
117
118
119
120
121
122
Residues
Wildtype
S-nitrosylation
E
300
200
100
0
-100
-200
-300
-400
Energy (kJ/mol)
584
585
586
587
588
Residues
Wildtype
S-nitrosylation

Supplementary Figure 13. Different MD simulation based analysis of EZH2-SUZ12 complex having either EZH2 WT and EZH2 S-nitrosylated form of the protein. (A) MD analysis using root mean square fluctuation (RMSF) to determine the flexibility of protein residues for both WT and S-nitrosylated SAL (region of EZH2) and SUZ-12. The RMSF value of both S-nitrosylated and WT residues remained relatively stable with a nearly similar trend, whereas the residues near the SAL and long loop region (residue 112-121 and 345-421) displayed significant fluctuations. (B) H-bond contact formed between residues 112-121 of SAL and 584-588 of SUZ12 over the course of 1μs MD simulation. (C) MMPBSA binding free energy. (D) Binding energy per residue of 112-121 of EZH2, (E) Binding energy per residue of 584-588 of SUZ12.

Supplementary Table 1. Contribution of individual interaction components and binding free energy for the EZH2-SUZ12 Complex.

Compounds	Van-der Waal energy (kJ/mol)	Electrostatic energy (kJ/mol)	Polar solvation energy (kJ/mol)	SASA energy (kJ/mol)	Binding energy (kJ/mol)
Wildtype	-1630 ± 62	-7099 ± 618	4275 ± 332	-196 ± 10	-4649 ± 454
S-nitrosylation	-1657 ± 76	-6642 ± 706	3923 ± 351	-206.485 ± 7	-4583 ± 453
