# Supplementary material for: S-nitrosylation of EZH2 alters PRC2 assembly, methyltransferase activity, and EZH2 stability to maintain endothelial homeostasis
Source: Nat Commun. 2025 Apr 27;16:3953. doi: 10.1038/s41467-025-59003-x (PMC12034783; doi:10.1038/s41467-025-59003-x)
Supplement: Supplementary file 3 — Description of Additional Supplementary Files [file 41467_2025_59003_MOESM3_ESM.pdf]

Supplementary Data 1*: List of all unique proteins associated with EZH2 in untreated (control) HUVEC as detected by MS analysis of EZH2 immunoprecipitates in two independent biological replicates. The enrichment was calculated based on the spectral count.*

Supplementary Data 2*: List of all unique proteins associated with EZH2 in treated (GSNO 100 μM) HUVEC as detected by MS analysis of EZH2 immunoprecipitates in two independent biological replicates. The enrichment was calculated based on the spectral count.*

Supplementary Data 3: *List of reagents and resources used in this study.*
