# Supplementary material for: S-nitrosylation of EZH2 alters PRC2 assembly, methyltransferase activity, and EZH2 stability to maintain endothelial homeostasis
Source: Nat Commun. 2025 Apr 27;16:3953. doi: 10.1038/s41467-025-59003-x (PMC12034783; doi:10.1038/s41467-025-59003-x)
Supplement: Supplementary file 7 — Reporting Summary [file 41467_2025_59003_MOESM7_ESM.pdf]

nature portfolio

Corresponding author(s): Syamantak Majumder

Last updated by author(s): Mar 15, 2025

Reporting Summary

Nature Portfolio wishes to improve the reproducibility of the work that we publish. This form provides structure for consistency and transparency in reporting. For further information on Nature Portfolio policies, see our Editorial Policies and the Editorial Policy Checklist.

Statistics

For all statistical analyses, confirm that the following items are present in the figure legend, table legend, main text, or Methods section.

n/a	Confirmed	
☐	☒	The exact sample size (*n*) for each experimental group/condition, given as a discrete number and unit of measurement
☐	☒	A statement on whether measurements were taken from distinct samples or whether the same sample was measured repeatedly
☐	☒	The statistical test(s) used AND whether they are one- or two-sided<br>*Only common tests should be described solely by name; describe more complex techniques in the Methods section.*
☒	☐	A description of all covariates tested
☒	☐	A description of any assumptions or corrections, such as tests of normality and adjustment for multiple comparisons
☐	☒	A full description of the statistical parameters including central tendency (e.g. means) or other basic estimates (e.g. regression coefficient) AND variation (e.g. standard deviation) or associated estimates of uncertainty (e.g. confidence intervals)
☐	☒	For null hypothesis testing, the test statistic (e.g. *F*, *t*, *r*) with confidence intervals, effect sizes, degrees of freedom and *P* value noted<br>*Give P values as exact values whenever suitable.*
☒	☐	For Bayesian analysis, information on the choice of priors and Markov chain Monte Carlo settings
☒	☐	For hierarchical and complex designs, identification of the appropriate level for tests and full reporting of outcomes
☒	☐	Estimates of effect sizes (e.g. Cohen's *d*, Pearson's *r*), indicating how they were calculated

Our web collection on statistics for biologists contains articles on many of the points above.

Software and code

Policy information about availability of computer code

Data collection	GPS-SNO prediction tools to determine the S-nitroylation sites,<br>Carl Zeiss Zen Blue for image acquisition using Confocal Laser Scanning Microscope (#LSM 880, Carl Zeiss) or Zeiss ApoTome 2.0 microscope,<br>Image Lab 6.0 for acquisition of immunoblots using Bio-Rad Chemidoc system<br>CytExert 2.4 for flow cytometry data Collection using the CytoFLEX flow cytometer (Beckman Coulter)
Data analysis	GROMACS 5.1.5, GraphPad Prism 9.5.1 and Prism 10, Proteome Discoverer (v2.5), ImageJ 1.52a or older

For manuscripts utilizing custom algorithms or software that are central to the research but not yet described in published literature, software must be made available to editors and reviewers. We strongly encourage code deposition in a community repository (e.g. GitHub). See the Nature Portfolio guidelines for submitting code & software for further information.

Data

Policy information about availability of data

All manuscripts must include a data availability statement. This statement should provide the following information, where applicable:

- Accession codes, unique identifiers, or web links for publicly available datasets
- A description of any restrictions on data availability
- For clinical datasets or third party data, please ensure that the statement adheres to our policy

Source data are provided with this paper. We have used PDB Code: 5HYN (https://doi.org/10.2210/pdb5HYN/pdb) for modeling EZH2 and SUZ12 complex to perform MD simulation study. The raw proteomics data has been deposited to the ProteomeXchange CONSORTIUM via the PRIDE partner repository with the dataset identifier PXD050209 (https://www.ebi.ac.uk/pride/archive/projects/PXD050209).

Research involving human participants, their data, or biological material

Policy information about studies with human participants or human data. See also policy information about sex, gender (identity/presentation), and sexual orientation and race, ethnicity and racism.

Reporting on sex and gender	Not Applicable
Reporting on race, ethnicity, or other socially relevant groupings	Not Applicable
Population characteristics	Not Applicable
Recruitment	Not Applicable
Ethics oversight	Not Applicable

Note that full information on the approval of the study protocol must also be provided in the manuscript.

Field-specific reporting

Please select the one below that is the best fit for your research. If you are not sure, read the appropriate sections before making your selection.

☒ Life sciences ☐ Behavioural & social sciences ☐ Ecological, evolutionary & environmental sciences

For a reference copy of the document with all sections, see nature.com/documents/nr-reporting-summary-flat.pdf

Life sciences study design

All studies must disclose on these points even when the disclosure is negative.

Sample size	We have carried out the experiments based on biological replicates. Unless otherwise specified, a minimum of three biological replicates were carried out for each experiments to perform statistical analysis.
Data exclusions	We have not excluded any data from the present study.
Replication	All experiments were replicated at least three times independently thereby achieving a minimum of three biological replicates, unless otherwise specified for specific experiments. In many cases, technical replicates were also carried out to ascertain the changes observed.
Randomization	We have randomly allocated the samples or animals used for the study to perform the experiments and end parameter analysis. For experiments involving diabetic animals, rats exhibiting high blood glucose level were allocated under diabetic group for long term study and analysis.
Blinding	All experiments carried for the current manuscript were blinded to group allocation during data collection and/or analysis.

Reporting for specific materials, systems and methods

We require information from authors about some types of materials, experimental systems and methods used in many studies. Here, indicate whether each material, system or method listed is relevant to your study. If you are not sure if a list item applies to your research, read the appropriate section before selecting a response.

Materials & experimental systems

n/a	Involved in the study
[ ]	[x] Antibodies
[ ]	[x] Eukaryotic cell lines
[x]	[ ] Palaeontology and archaeology
[ ]	[x] Animals and other organisms
[x]	[ ] Clinical data
[x]	[ ] Dual use research of concern
[x]	[ ] Plants

Methods

n/a	Involved in the study
[x]	[ ] ChIP-seq
[ ]	[x] Flow cytometry
[x]	[ ] MRI-based neuroimaging

Antibodies

Antibodies used

EZH2 mAb Cell Signaling Technology #5246 AB_2798987 WB(1:1000), IP(1μg), IF(1:1000), IF/IHC(1:100)
Jarid 2 Rabbit mAb Cell Signaling Technology #13594 AB_2798269 WB(1:1000)
SUZ12 Rabbit mAb Cell Signaling Technology #3737 AB_2799833 WB(1:1000)
EZH1 Rabbit mAb Cell Signaling Technology #42088 AB_2799212 WB(1:1000)
EED Rabbit mAb Cell Signaling Technology #51673 AB_2923355 WB(1:1000)
AEBP2 Rabbit mAb Cell Signaling Technology #14129 AB_2798398 WB(1:1000)
H3K27me3 Rabbit mAb Cell Signaling Technology #9733 AB_2797834 WB(1:1000), cut&run(1:50), IF(1:1000), IF/IHC(1:100)
UTX Rabbit mAb Cell Signaling Technology #33510 AB_2721244 WB(1:1000)
JMJD3 Rabbit mAb Cell Signaling Technology #3457 AB_1549620 WB(1:1000)
HA-Tag Rabbit mAb Cell Signaling Technology #3724 AB_2665471 WB(1:1000), IP(1μg)
GAPDH Rabbit mAb Cell Signaling Technology #5174 AB_2799390 WB(1:1000)
α-Tubulin Mouse mAb Abclonal AC012 AB_2768341 WB(1:1000)
β-actin Rabbit mAb Cell Signaling Technology #3700 AB_2566811 WB(1:1000)
H3 Rabbit mAb Cell Signaling Technology #4499 AB_2943236 WB(1:1000)
S-Nitrocysteine Mouse mAb Abcam #94930 AB_10697568 WB(1:1000), IP(1μg)
Ubiquitin Rabbit pAb Cell Signaling Technology #3933 AB_3075532 WB(1:1000)
eNOS Rabbit mAb Cell Signaling Technology #32027 AB_2728756 WB(1:1000)
ICAM 1 Rabbit mAb Cell Signaling Technology #4915 AB_2280018 WB(1:1000)
VE-cadherin Mouse mAb Santa Cruz sc-9989 AB_2077957 WB(1:1000), IF(1:1000), IF/IHC(1:25)
TMT Antibody Mouse mAb Thermo Fisher Scientific 90075 AB_10854708 WB(1:1000), IP(1μg)
14-3-3 epsilon Rabbit mAb Abclonal A4933 AB_2863392 WB(1:1000)
Anti-Rabbit IgG (H+L), HRP Conjugate antibody Cell Signaling Technology #7074 AB_2099233 WB(1:2000)
Anti-Mouse IgG (H+L), HRP Conjugated antibody Cell Signaling Technology #7076 AB_330924 WB(1:2000)
Goat anti-Rabbit IgG (H+L) Cross-Adsorbed Secondary Antibody, Alexa Fluor™ 488 Thermo Fisher Scientific # A32723 AB_143165 IF (1:4000)
Goat anti-Rabbit IgG (H+L) Highly Cross-Adsorbed Secondary Antibody, Alexa Fluor™ 546 Thermo Fisher Scientific # A-11035 AB_2534093 IF(1:4000)
HA Tag Monoclonal Antibody (2-2.2.14), Alexa Fluor™ 647 Thermo Fisher Scientific # 26183-A647 AB_2610626 IF(1:2000)

Validation

Each of the antibodies used in the study were validated by the commercial source. No in-house or previously unpublished antibodies were used in the study. The catalog numbers and associated details are provided in the Methods sections as well as Supplementary Data S3 file.

Eukaryotic cell lines

Policy information about cell lines and Sex and Gender in Research

Cell line source(s)

EA.hy926 ATCC, Manassas, USA #CRL-2922
HUVEC HiMedia Laboratories #RM1112
HEK-293 Kind gift from Prof. Uma Dubey (Department of Biological Sciences, Birla Institute of Technology and Science Pilani, Pilani Campus, India) Originally Procured from NCCS Pune, India

Authentication

Cell lines have been authenticated by the supplier prior to delivery. Upon receiving, we confirm the expression of cell specific markers using immunoblot techniques to validate the cell type under study.
For EA.hy926 cells please check the supplier website https://www.atcc.org/products/crl-2922
For HUVEC please check supplier website https://www.himedialabs.com/in/hifitm-human-umbilical-vein-endothelial-cells-huvec-single-donor.html

Mycoplasma contamination

The cell lines were mycoplasma free.

Commonly misidentified lines (See ICLAC register)	No misidentified cell lines were used in the current study.

Animals and other research organisms

Policy information about studies involving animals; ARRIVE guidelines recommended for reporting animal research, and Sex and Gender in Research

Laboratory animals	Both Male Sprague Dawley (SD) and Male Wistar Rats were bred at Breeding Facility of Animal Housing, BITS Pilani, Pilani Campus Originally Procured from National Laboratory Animal Centre, CSIR-CDRI, Lucknow, India
Wild animals	No wild animal are used in the study.
Reporting on sex	Only Male Wistar and SD rats are used in the present study
Field-collected samples	No field collected samples were used.
Ethics oversight	All animal studies were carried out according to the Committee for Control and Supervision of Experiments on Animals (CCSEA) guidelines for the use of laboratory animals. All the experimental procedure involving rodent studies were reviewed and approved by the Institutional Animal Ethics Committee (IAEC) of BITS Pilani, Pilani Campus and was in accordance with the protocol approved by IAEC, BITS-Pilani (Protocol Approval No: IAEC/RES/31/13/REV-1/33/19, and Protocol Approval No: IAEC/RES/23/05/Rev-1/28/27).

Note that full information on the approval of the study protocol must also be provided in the manuscript.

Plants

Seed stocks	Not Applicable
Novel plant genotypes	Not Applicable
Authentication	Not Applicable

Flow Cytometry

Plots

Confirm that:

- [x] The axis labels state the marker and fluorochrome used (e.g. CD4-FITC).
- [x] The axis scales are clearly visible. Include numbers along axes only for bottom left plot of group (a 'group' is an analysis of identical markers).
- [x] All plots are contour plots with outliers or pseudocolor plots.
- [x] A numerical value for number of cells or percentage (with statistics) is provided.

Methodology

Sample preparation	Dead Cell Apoptosis Kit with Annexin V FITC and propidium iodide (PI) for flow cytometry (#V13242, Thermo Fisher Scientific) was employed to evaluate cellular apoptosis of EC exposed to SNP (500 µM) for 24 hours. EA.hy926 cells were treated with SNP and further processed following manufacturer protocol. Briefly, the cells were harvested after the incubation period and washed twice in cold PBS. Following centrifugation, the cell pellet was resuspended in 1X annexin-binding buffer (approx. 1× 106cell/ml), and 5 µl of FITC annexin V (component A) and 1 µl of (PI) (100 µg/ml) were added to each 100 µl of cell suspension. After 15 minutes incubation at room temperature, the cell suspension was further diluted with 400 µl PBS and the stained cells were analyzed
Instrument	CytoFLEX flow cytometer (Beckman Coulter)
Software	CytExert 2.4
Cell population abundance	We have taken at least 10,000 cells for performing this analysis for each experimental groups including control and SNP treated cells.

Gating strategy	FSC and SSC are set such that to remove debris and doublets and further to identify the cell population of interest through the Annexin V and Propidium Iodide staining to distinguish between viable, early apoptotic, late apoptotic, and necrotic cells. We have showed the gating strategy in the first panel of Figure S1D showing SSC and FSC in one of the biological replicate.

☒ Tick this box to confirm that a figure exemplifying the gating strategy is provided in the Supplementary Information.
